# Higher Isolation of NDM*-*1 Producing *Acinetobacter baumannii* from the Sewage of the Hospitals in Beijing

**DOI:** 10.1371/journal.pone.0064857

**Published:** 2013-06-03

**Authors:** Chuanfu Zhang, Shaofu Qiu, Yong Wang, Lihua Qi, Rongzhang Hao, Xuelin Liu, Yun Shi, Xiaofeng Hu, Daizhi An, Zhenjun Li, Peng Li, Ligui Wang, Jiajun Cui, Pan Wang, Liuyu Huang, John D. Klena, Hongbin Song

**Affiliations:** 1 Institute of Disease Control and Prevention, Academy of Military Medical Science, Beijing, People’s Republic of China; 2 State Key Laboratory for Infectious Disease Prevention and Control, China Center of Disease Control and Prevention, Beijing, People’s Republic of China; 3 United States Centers for Disease Control and Prevention, China –US Collaborative Program on Emerging and Re-emerging Infectious Diseases, Beijing, People’s Republic of China; Rockefeller University, United States of America

## Abstract

Multidrug resistant microbes present in the environment are a potential public health risk. In this study, we investigate the presence of New Delhi metallo-β-lactamase 1 (NDM-1) producing bacteria in the 99 water samples in Beijing City, including river water, treated drinking water, raw water samples from the pools and sewage from 4 comprehensive hospitals. For the *bla*_NDM_-1 positive isolate, antimicrobial susceptibility testing was further analyzed, and Pulsed Field Gel Electrophoresis (PFGE) was performed to determine the genetic relationship among the NDM-1 producing isolates from sewage and human, as well as the clinical strains without NDM-1. The results indicate that there was a higher isolation of NDM-1 producing *Acinetobacter baumannii* from the sewage of the hospitals, while no NDM-1 producing isolates were recovered from samples obtained from the river, drinking, or fishpond water. Surprisingly, these isolates were markedly different from the clinical isolates in drug resistance and pulsed field gel electrophoresis profiles, suggesting different evolutionary relationships. Our results showed that the hospital sewage may be one of the diffusion reservoirs of NDM-1 producing bacteria.

## Introduction

The increasing threat of antibiotic resistance in microbes affecting humans has been recognized as a challenge for treatment of clinical infection. The emergence and spread of pathogenic bacteria with broad spectrum antibiotic resistance pose real threats to the public health systems of any country. In 2009, a new metallo-β-lactamase gene (*bla*NDM*-*1), encoding the metallo-β-lactamase protein New Delhi metallo-β-lactamase 1 (NDM-1) with high carbapenemase activity and which can destroy carbapenem-type antibiotics, was first identified from a Swedish patient of Indian origin [1]. Carbapenems represent a last line of antibiotic defense for many infections; resistant organisms are capable of causing death in infected hosts. To date, infections associated with NDM-1 positive strains have been reported in several countries and district including U.K., U.S., Canada, Australia, France, Holland, India and China, Sweden, Sultanate of Oman, Kenya, Singapore, Bangladesh, Australia, Switzerland, France, Iraq, Norway, Singapore, Belgium, Montenegro, Germany, Pakistan, Italy, Japan, Spain [2]. In total, strains of multiple speciesof *bla*NDM-1 carrying bacteria, including *Shigella boydii* and *Vibrio cholerae,* have been identified worldwide [2]–[4]. The genes encoding NDM-1 are known to be carried on a plasmid, and it is suspected horizontal gene transfer (HGT) promotes the exchange of resistance among Gram-negative organisms.

Sewage is a complex matrix composed of multiple components from many fecal sources. Discharged sewage from hospitals if improperly treated may contain pathogens and antibiotic residues that could lead to acute infections, or to the selection and spread of resistance through the HGT of genetically mobile resistance cassettes. Sewage is a hot spot of gene transfer between organisms [5]. In this way, the hospital may become a source of spread of the resistant bacteria. NDM-1 producing bacteria have been identified in water pools, sewage and tap water in New Delhi [4].

*Acinetobacter baumannii* is an important pathogen for hospital-acquired infections and widely distributed in a variety of environments in the hospital. Infections caused by *A. baumannii* expressing a broad drug-resistance spectrum have been frequently reported worldwide. The resistance patterns associated with the isolates has created great obstacles for clinical treatment. Additionally, strains are resistant to heat, ultraviolet and chemical sanitizers and thus not easily disinfected by routine sanitizers. Recently, 14% of *bla*NDM-1 carrying bacteria and many *A. junii* isolates and a couple of *A.baumanii* isolates were found from over 10,000 faecal samples in China [6]. Two *Acinetobacter johnsonii* strain carrying a blaNDM-1 plasmid was also isolated from Sewage of a hospital in China [7]. In addition, it was proved that NDM-1 is a recently made gene via the fusion of two resistance genes and that this event happened in Acinetobacter [8]. Beijing, the capital city of China, has an estimated population of 20 million, spread over a 16,410.54 km^2^ area. Potable water is a limited resource to Beijing, therefore it is of prime importance to ensure the water systems of Beijing City remain pathogen-free. Surveillance of water systems that may be adversely affected by point sources of contamination, such as hospital effluent, is critical to the maintenance of safe water supplies. The aims of this study were to determine whether NDM-1 producing *A. baumannii* could be detected in hospital effluent, environmental sources, and clinical cases in Beijing and to determine the relationship between NDM-1 and non-NDM-1 producing *A. baumannii* isolates regardless of their source of origin.

## Materials and Methods

### Collection and Identification of Bacteria

The institutional review board of the Academy of Military Medical Sciences waived the need for written informed consent from the participants. This study is approved and authorized for each location by the Academy of Military Medical Sciences Review Board. There was no request for a specific permission according to Chinese law. A total of 119 water samples were gathered from river water (*n* = 20), treated drinking water samples (*n* = 50), sewage from 4 comprehensive hospitals (*n* = 20), raw water samples from the pools (*n* = 9) and community life sewage (*n* = 20) in Beijing from September to November 2010. For each sample, 500 ml of target water was collected and a 100 ml aliquot was centrifuged at 1000×*g* for 10 minutes at ambient temperature. After carefully decanting the supernatant, pellets were re-suspended with 1 ml Luria-Bertani (LB) liquid medium and 400 µl samples were seeded onto LB agar plates containing imipenem (10 µg/ml). All colonies on the culture plates were selected and identified by PCR as previously described [4] and was further sequenced for confirmation. All NDM-1 positive strains were identified using Vitek GNI+ cards (bioMérieux, France), and sequence analysis of the 16S rRNA gene. The primers used to amplify the 16s rRNA gene were 5′-TACCTTGTTACGACTT-3′ and 5′- AGAGTTTGATCITGGA-3′ [9]. The other *A. baumannii* isolates, as identified by Vitek GNI+ cards (bioMérieux, France) were isolated in our laboratory, between September 2009 and February 2010 (Table 1).

**Table 1:** *A. baumannii* isolates with or without *bla*NDM-1 from the environment, sewage and human.

Isolate	Source	Region	NDM-1	isolation by year
HHG8<$>\raster(65%)="rg2"<$>, HHG8<$>\raster(65%)="rg3"<$>	Environment	Beijing	+	2009
WJ3-2, WJ3-5, WJ0117, WJ0111, WJ0102<$>\raster(65%)="rg1"<$>, WJ0135, WJ0102<$>\raster(65%)="rg2"<$>,WJ0102, WJ0147, 3070341	Sewage	Beijing	+	2010
10051750green	Human	Xiamen	+	2009
44, 65, ICU-1, Evn-60, 25, Evn-59,	Human	Beijing	_	2009
10051442blue, 192, 270, 136, 372,104,	Human	Xiamen	_	2009
NJ35, NJ35-1, NJ87-1-1,	Human	Nanjing	_	2009
10092903, 10092908, 10092901, 10092902, 10092904, 10092907, 10092910, Evn-52, Evn-37, Evn-38, Evn-50, Evn-41, Evn-47,Evn-43, Evn-44	Environment	Beijing	_	2009

### Antibiotic Susceptibility Testing

Bacterial susceptibility testing was carried out by the Kirby-Bauer method according to the Clinical Laboratory Standards Institute (CLSI) guidelines (2008) [10]. The antibiotic discs used were imipenem, meropenem, cefdazadime, cefotaxime, cefepime, gentamicin, tobramycin, tetracycline, ciprofloxacin, polymyxin B, chloramphenicol and tigecycline. The tested bacterium was picked up with sterile loop and suspended in peptone water and incubated at 37°C for 3 hours. The turbidity of the suspension was adjusted to 0.5 McFarland’s standard, and the suspension was then spread on the surface of a LB agar plates using sterile cotton swab. The antimicrobial susceptibility test disc was placed on the agar. The plates were incubated at 37°C overnight. The zone of inhibition was measured and interpreted as per the CLSI guidelines.

### Conjugation and Transformation Experiments and Plasmid Analysis

Conjugation transfer assay was performed in broth culture with *E. coli* J53 as the recipient. Donor was respectively mixed at a ratio of 1∶3 with ten *A. baumannii* containing the *bla*NDM-1 gene from the sewage. Transconjugants were selected on MacConkey medium containing sodium azide (100 mg/mL) and ceftazidime (16 mg/mL). Plasmid DNA was respectively extracted from ten *A. baumannii* containing the *bla*NDM-1 gene from the sewage, and was transformed by electroporation into competent *E. coli* JM109. The transformants were selected on LB agar plates containing ceftazidime (16 mg/mL). Transformants and transconjugants were further confirmed by Vitek GNI+ cards (bioMérieux, France) and tested for antimicrobial susceptibility by the Kirby-Bauer method according to CLSI (2008). Plasmids were extracted from donor strain, recipient strain (*E. coli* J53 and *E. coli* JM109), transconjugant and transformant by the QIAGEN Large-Construct Kit and were further analyzed by specific PCR and sequencing for blaDNM-1.

### PFGE

Pulsed-field gel electrophoresis (PFGE) of *bla*NDM-1 carrying *A. baumannii* isolates recovered from sewage, environmental sources, and clinical isolates with or without *bla*NDM-1 was carried out with a CHEF-Mapper XA PFGE system (Bio-Rad, USA) for 22 h at 6 V/cm and 14°C, with a pulse angle of 120° and pulse times from 5 to 20 s. The restriction endonuclease ApaI was used for in-situ digestion of intact *A. baumannii* genomic DNA. PFGE banding patterns were analyzed visually by using a Bio-Rad Gel Doc 2000 system. Genetic relationship was analyzed by the BioNumerics version 46.0 software.

## Results

### Higher NDM*-*1 Producing *A. baumannii* Isolation from the Sewage of the Hospitals

Ten isolates containing the *bla*NDM-1 gene were identified in sewage prior to disinfect from the general hospitals; this included an effluent sample from a hospital after disinfecting by chlorination before discharge and community life sewage (Table 2). No NDM*-*1 producing isolates were recovered from samples obtained from the river, drinking, or fishpond water and community life sewage. Biochemical identification and sequencing of 16S rRNA demonstrated that the ten isolates were *A*. *baumannii*.

**Table 2:** Recovery of carbapenem-resistant and *bla*NDM-1 positive *A. baumannii* isolates from sewage sources of four Beijing hospitals and community.

Sample ID	disinfection	Sample number	Carbapenem resistant isolates^a^	*bla*NDM-1 positive isolates^b^
A	Before disinfection	4	32	3
	After disinfection	1	5	1
B	Before disinfection	4	29	2
	After disinfection	1	3	0
C	Before disinfection	4	41	1
	After disinfection	1	1	0
D	Before disinfection	4	37	3
	After disinfection	1	2	0
E^c^	Before treatment	20	37	0

a Phenotypic resistance to carbapenem.

b A. baumannii only.

A,B,C,D: The hospital sewage; E: Community life sewage.

### Characterization of the Drug Resistance Profile of the *bla*NDM-1 Positive Isolates

Antimicrobial susceptibility testing was performed to determine the drug resistance profile of the *bla*NDM-1 positive isolates. Previous studies indicated that most clinical NDM-1 producing bacteria were resistant to all antibiotics except colistin and tigecycline [1], [3]. Zhou et al [11] reported clinical isolates from a child patient in China were resistant to all β-lactams except aztreonam but sensitive to aminoglycosides and quinolones. In this study, they were sensitive to aminoglycosides, chloramphenicol, colistin and tigecycline that *A. baumannii* possessing *bla*NDM-1 were isolated from the hospital environment and the hospital sewage (Table 3), In addition, two isolates were sensitive to quinolones and six isolates were non-resistant (four susceptible and two intermediate) to tetracycline, respectively. The data indicates that sewage-associated isolates of *A. baumannii* have unique antibiotic resistance profiles from those reported for clinically-obtained *A. baumannii* harbouring *bla*NDM-1.

**Table 3:** Antibiotic resistance profiles of *A. baumannii* isolates carrying *bla*NDM-1 isolated from sewage.

Strains	Drug resistance§											
	IPM	MEC	CAZ	CTX	FEP	GM	TM	TE	CIP	PB	CHL	TGC
WJ0135	R	R	R	R	R	S	S	S	S	S	S	S
WJ0117	R	R	R	R	R	S	S	S	S	S	S	S
HHG8<$>\raster(65%)="rg2"<$>	R	R	R	R	R	S	S	S	S	S	S	S
HHG8<$>\raster(65%)="rg3"<$>	R	R	R	R	R	S	S	S	S	S	S	S
3070341	R	R	R	R	R	S	S	I	S	S	S	S
WJ0102<$>\raster(65%)="rg1"<$>	R	R	R	R	R	S	S	I	S	S	S	S
WJ0102<$>\raster(65%)="rg2"<$>	R	R	R	R	R	S	S	R	S	S	S	S
WJ0111	R	R	R	R	R	S	S	R	S	S	S	S
WJ0147	R	R	R	R	R	S	S	R	S	S	S	S
WJ0102	R	R	R	R	R	S	S	R	S	S	S	S
WJ3-2	R	R	R	R	R	S	S	S	R	S	S	S
WJ3-5	R	R	R	R	R	S	S	S	R	S	S	S

Abbreviations: IPM: imipenem (10 µg); MEM: meropenem (10 µg); CAZ: cefdazadime (30 µg); CTX: cefotaxime (30 µg); FEP: cefepime (30 µg); CN: gentamicin (10 µg); TOB: tobramycin (10 µg); TE: tetracycline (30 µg); CIP: ciprofloxacin (5 µg); PB: polymyxin B (300U); CHL: chloramphenicol (30 µg); TGC: tigecycline (15 µg); R: resistance; S: sensitivity; I: intermediate;

§ According to CLSI guidelines.

### Plasmid Analysis

**Figure 1:**
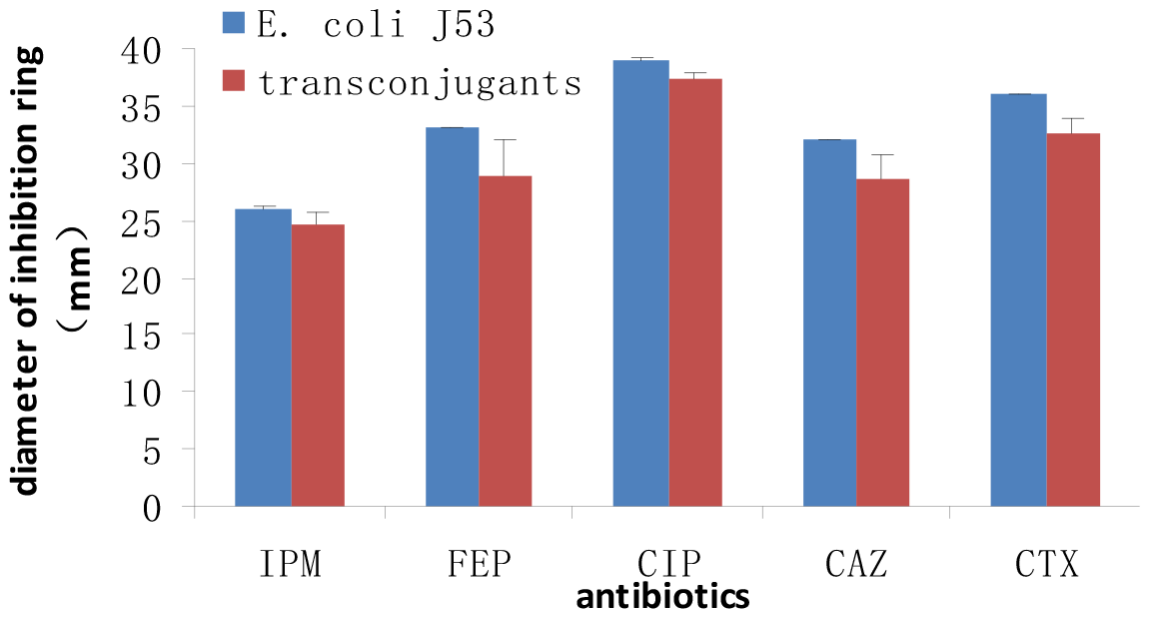
Antibiotic susceptibility testing of recipient strain *E. coli* J53 and transconjugants. IPM: imipenem; FEP: cefepime; CIP: ciprofloxacin; CAZ: cefdazadime; CTX: cefotaxime; The diameter of the conjugant to all the antibiotics reduced significantly, P<0.05.

The conjugation and transformation experiments were performed to investigate whether the gene for NDM-1 in *A. baumannii* isolates recovered from sewage were located on plasmids and whether the transconjugant and transformant reduced susceptibility of the recipient strain towards antibiotics. Transconjugants and transformants were respectively randomly chosen, and the *bla*NDM-1 gene was determined by PCR amplification and sequencing (see in Fig. S1). The result showed that the plasmids carrying blaNDM-1 from A. baumannii from the sewage were successfully transferred to E. coli J53 and *E. coli* JM109. Susceptibility tests revealed that both the transconjugant and transformant decreased susceptibility to imipenem, cefepime, ciprofloxacin, cefdazadime, cefotaxime as compared with the recipients *E. coli* J53 and JM109 (Figure 1). Interestingly, all the transconjugants and transformants stably maintained the *bla*NDM-1-containing plasmid after seven passages in the absence of selection pressure.

### PFGE Analysis

**Figure 2:**
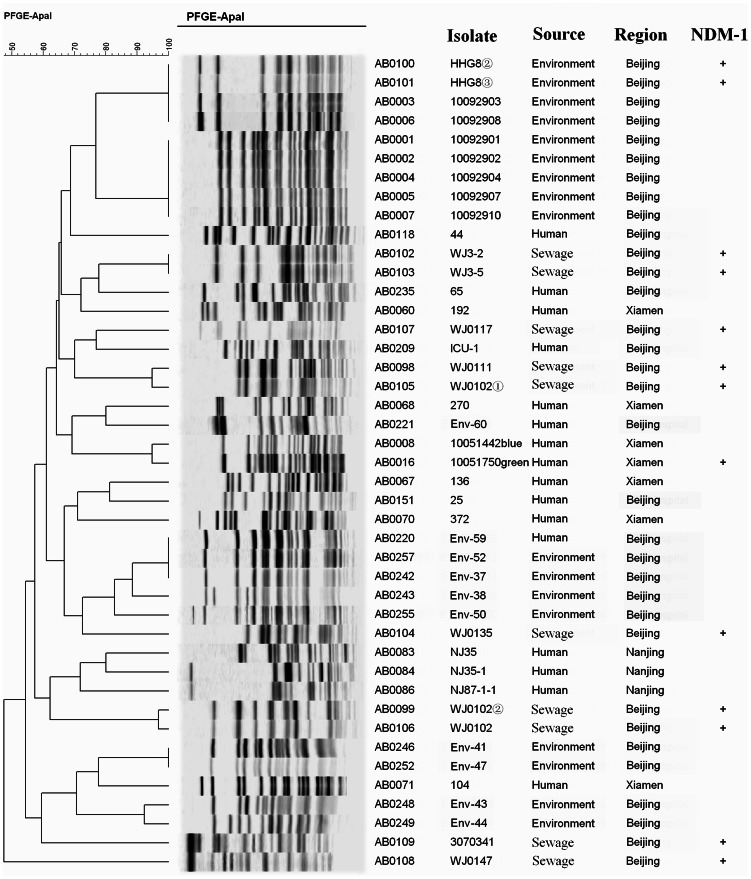
Dendrogram derived from PFGE patterns of ApaI-digested *A.* baumanii DNA. *A. baumannii* isolates were recovered from clinical cases (Beijing, Xiamen, and Nanjing) and the hospital environment (Beijing). Abbreviations: isolate: key number given to isolate in BioNumerics software (ABxxxx) and original isolate designation; source: location of recovery of isolate where environment could be (list sources here); region: city of isolation; NDM-1: “+” indicates an isolate containing *bla*NDM-1.

To investigate the genetic relationship between *bla*NDM-1 carrying *A*. *baumannii* isolates recovered from sewage, environmental sources, and clinical isolates with or without *bla*NDM-1, PFGE was performed as previously described [12]. Among the 13 *bla*NDM-1 positive isolates recovered in this study, 11 PFGE patterns were observed (Figure 2). Two isolates, WJ3-2 and WJ3-5, had an indistinguishable *Apa* I-PFGE pattern among the ten sewage-recovered isolates of *A. baumannii*; two additional pairs (WJ0102, WJ0102-2; and WH0111 and WJ0102-1) were ≥94% similar. All six of these isolates were *bla*NDM-1-positive and had nearly identical phenotypic antimicrobial sensitivity profiles (both WJ0111 and WJ0102-1 were TE non-susceptible, WJ0111 was resistant and WJ0102-1 was recorded as intermediate). Collectively this data suggests these isolates came from a common source. Similarly, environmental isolates grouped into several distinct clusters. Furthermore, a large number of band variations were observed between the *bla*NDM-1 carrying isolates recovered from hospital sewage, and the *bla*NDM-1 carrying clinical and hospital environmental isolates. This variation was also evident in *A. baumannii* isolates without *bla*NDM-1. These results suggest that *A. baumannii* isolates in Beijing hospital system are genetically diverse overall, and that among the *bla*NDM-1 carrying *A. baumannii* isolates, several clones were detected.

## Discussion

Sewage from hospitals has the potential to contain a large number of pathogens including parasite ova, pathogenic bacteria and viruses; therefore, sewage should be disinfected before discharge. Guardabassi et al [13] reported a large number of bacteria remained in the sewage discharged from the hospitals, antibiotic production factories and livestock farms after the disinfection process. We identified ten *bla*NDM-1 carrying isolates from the water samples collected from four general hospitals in Beijing, China. Even though waste water disinfection was performed in all hospitals, a *bla*NDM-1 carrying isolate was still recovered from the treated sewage. Sewage that is not treated appropriately may pollute the water systems including surface water, ground water and drinking water, which may enhance the possibility of infection induced by *bla*NDM-1 positive strains in human body. Additionally, the *bla*NDM-1 gene is known to be transferred between bacterial genera [3]. These observations suggest an increase exposure of hospitalized patients to *bla*NDM-1 carrying microbes and imply the incidence of *bla*NDM-1-associated infections in hospital-acquired infections, caused directly by *A. baumannii* or a bacterial genera that acquired the resistance mechanism from *A. baumannii* after mixing in a permissive environment such as sewage discharge from hospitals, is likely to increase in China.

As previously reported, NDM-1-producing bacteria are resistant to β-lactamase antibiotics [3]. The NDM-1 positive isolates associated with sewage in this study were all sensitive to aminoglycosides, chloramphenicol, colistin and tigecycline. In addition, two isolates were sensitive to quinolones and six isolates were non-resistant to tetracycline, respectively. Our study indicated differences in drug resistance profiles between *bla*NDM-1-associated isolates obtained from sewage and those previously reported from clinical isolates. With the wide and excessive using of antibiotics in clinic, clinical strains quickly evolved into multi-drug resistant (MDR) or pan-drug resistant (PDR) bacteria. It contains a certain amount of antibiotic residues in the sewage, but the concentration of antibiotic is relatively lower than that in the clinic, which has to a certain extent slowed down the speed of evolution of the environmental resistant strains, and result in environmental resistant strains are still sensitive to some antibiotics. This may be one of the reasons for these differences. Even though no associated infection has been reported to date from these hospitals in Beijing, we can not neglect the potential threats of the drug resistance gene to the ecosystem and human lives.

The plasmids carrying the *bla*NDM gene successfully transferred to the recipient *E. coli* J53 and *E. coli* JM109, suggesting that this plasmid is mobile. The transconjugant and transformant decreased susceptibility to imipenem, cefepime, ciprofloxacin, cefdazadime, cefotaxime, and stably maintained the *bla*NDM-1-containing plasmid of *A. baumannii*, which displays the potential for the spread of *bla*NDM through plasmid transmission from A. baumannii to Enterobacteriaceae in the natural environment. To analyze the genetic diversity and evolutionary relationships among isolates, PFGE was performed. The results further support the genetic diversity of the *bla*NDM-1 carrying *A. baumannii*. Therefore, we hope to raise the attention of *bla*NDM-1-associated bacteria in the water systems of Beijing City. Currently, it is not clear to the reason of the difference of the drug resistance and genetic relatedness among the *bla*NDM-1 carrying obtained isolates from the sewage and the clinic, further in-depth genetic analysis must be performed.

## Supporting Information

Figure S1 Identification of a mobile blaNDM-1 gene. Plasmids were extracted from donor strain, recipient strain *E. coli* J53 and *E. coli* JM109, transconjugants, transformants and PCR analysis of the blaNDM-1 gene. M: marker; 1-3: transconjugants; 4-5: transformants; 6: *E. coli* J53; 7: *E. coli* JM109; 8-9: WJ3-5, WJ0135. The experimental results of other *A. baumannii* containing the blaNDM-1 gene from the sewage were similar to those.
(TIF)
