# Supplementary figures and images for: Higher Isolation of NDM-1 Producing Acinetobacter baumannii from the Sewage of the Hospitals in Beijing
Source: PLoS One. 2013 Jun 3;8(6):e64857. doi: 10.1371/journal.pone.0064857 (PMC3670931; doi:10.1371/journal.pone.0064857)

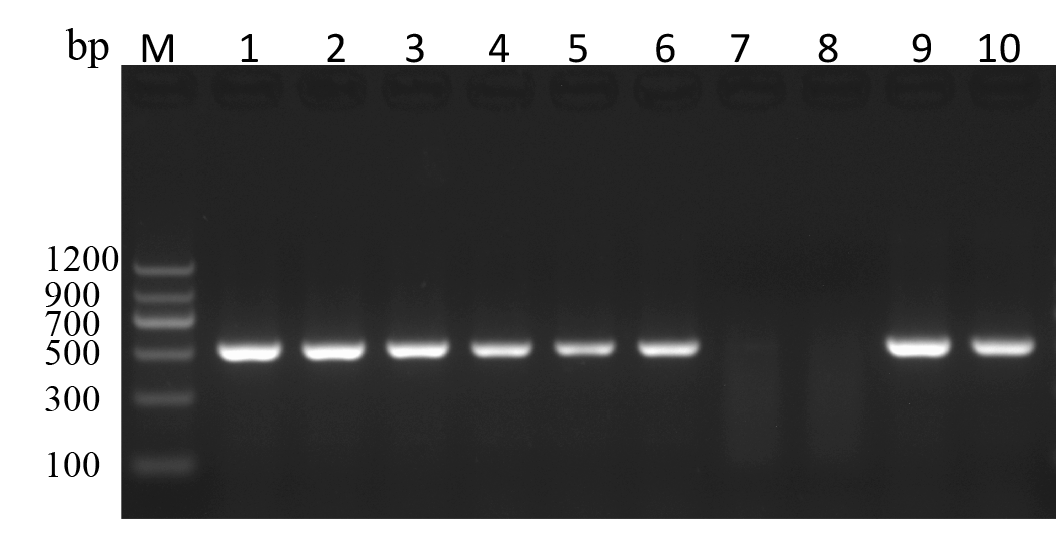

Supplement: Figure S1 — Identification of a mobile blaNDM-1 gene. Plasmids were extracted from donor strain, recipient strain E. coli J53 and E. coli JM109, transconjugants, transformants and PCR analysis of the blaNDM-1 gene. M: marker; 1-3: transconjugants; 4-5: transformants; 6: E. coli J53; 7: E. coli JM109; 8-9: WJ3-5, WJ0135. The experimental results of other A. baumannii containing the blaNDM-1 gene from the sewage were similar to those. (TIF) [file pone.0064857.s001.tif]
